# Research note: Development of a recombinant duck enteritis virus vector expressing DHAV-3 VP1 and DTMUV prM/TE genes

**DOI:** 10.1016/j.psj.2026.107501

**Published:** 2026-07-27

**Authors:** Wenfeng Jia, Zhi Wu, Aiqi Tan, Yuting Cheng, Qingkang Zhou, Shanyuan Zhu, Anping Wang

**Affiliations:** Jiangsu Key Laboratory for High-Tech Research and Development of Veterinary Biopharmaceuticals, Jiangsu Agri-Animal Husbandry Vocational College, 8 East Phoenix Road, Taizhou, Jiangsu, 225300, PR China

**Keywords:** Duck enteritis virus, CRISPR/Cas9 genome editing, Duck hepatitis A virus, Duck Tembusu virus, Viral vector

## Abstract

Duck enteritis virus (**DEV**) is a promising viral vector for vaccine development. In a previous study, an HDR-CRISPR/Cas9-based strategy was used to generate a recombinant virus, rDEV-DHAV-VP1, by inserting the VP1 gene of duck hepatitis A virus type 3 (**DHAV-3**) into the UL27/UL26 intergenic region of DEV vaccine strain, resulting in good genetic stability and immunogenicity. In the present study, the same strategy was applied to insert the EGFP gene into the US7/US8 and LORF11/UL55 intergenic regions of a DEV vaccine strain. Among the evaluated insertion sites, the highest level of EGFP expression was observed at the US7/US8 locus, followed by the UL27/UL26 locus. Based on rDEV-DHAV-VP1, the pre-membrane (**prM)** and truncated envelope (**TE**) genes of duck Tembusu virus (**DTMUV**) were further inserted into the US7/US8 locus, resulting in a bivalent recombinant virus, rDEV-VP1-prM/TE. The recombinant virus exhibited growth kinetics comparable to those of the parental virus, while maintaining efficient expression and high genetic stability of the inserted genes. These findings indicate that the HDR-CRISPR/Cas9 system is an efficient strategy for generating stable DEV-based recombinant vectors and provides a promising platform for the development of multivalent vaccines against major duck viral diseases.

## Introduction

Duck enteritis virus (**DEV**), also known as duck plague virus (**DPV**), is a member of the family *Herpesviridae* characterized by a large, stable double-stranded DNA genome containing multiple non-essential regions that can accommodate foreign gene insertion (Dhama et al., 2017). Owing to its restricted host range, favorable biosafety profile, and ability to induce robust humoral and cellular immune responses, DEV has emerged as a promising viral vector for avian vaccine development (Wang et al., 2024). With the advancement of genome editing technologies, particularly CRISPR/Cas9-mediated homology-directed repair (HDR), the efficiency and precision of recombinant DEV construction have been markedly improved compared with conventional homologous recombination approaches (Jia et al., 2025). However, the selection of optimal genomic insertion sites remains a critical factor influencing the expression level and stability of foreign antigens, which directly affects the immunogenicity of recombinant vaccines.

Duck hepatitis A virus (**DHAV**) and duck Tembusu virus (**DTMUV**) are two major viral pathogens responsible for significant economic losses in the duck industry worldwide (Li et al., 2025). The VP1 protein of DHAV is a major structural protein containing key neutralizing epitopes, whereas the pre-membrane (**prM)** and envelope (**E**) proteins of DTMUV are essential for viral assembly and induction of protective immune responses (Chen et al., 2014; Truong and Cheng, 2022); notably, the truncated E protein (**TE**) exhibits improved immunogenicity (Liu et al., 2019; Li et al., 2020).

In this study, we employed an HDR-CRISPR/Cas9-based strategy to systematically evaluate potential insertion loci in the DEV genome and subsequently constructed a recombinant DEV expressing DHAV VP1 and DTMUV prM/TE genes, aiming to develop an efficient DEV-based multivalent vaccine platform.

## Materials and methods

### Construction of plasmids and recombinant viruses

gRNA plasmids, donor plasmids, and recombinant duck enteritis viruses (**DEV**) were constructed as previously described (Jia et al., 2026). Guide RNAs (**gRNAs**) targeting the DEV genome were designed for the LORF11/UL55 locus (5′-CAGTGAACGCTGAACAAGCTAGG-3′) and the US7/US8 locus (5′-ATGAGGTAATAGGCTATGGGTGG-3′), and cloned into the pX459 vector to generate gRNA-Cas9 expression plasmids.

Donor plasmids contained approximately 1000 bp homologous arms flanking the target insertion sites. Foreign genes were assembled as expression cassettes driven by the CMV promoter and terminated by the bovine growth hormone (**BGH**) polyadenylation signal. The pre-membrane (**prM)** and truncated envelope (**TE**) genes of duck Tembusu virus (**DTMUV**) were codon-optimized for duck, with a Flag tag fused at the N-terminus and an EGFP reporter fused at the C-terminus. The TE gene corresponds to amino acids 1-451 of the DTMUV envelope (**E**) protein.

Recombinant rDEV-EGFP was generated using the DEV vaccine strain (**DEV-Vac**) as the parental backbone, and rDEV-VP1-prM/TE was subsequently constructed based on rDEV-DHAV-VP1. Recombinant viruses were obtained using a transfection-infection strategy, in which CEF cells were co-transfected with gRNA-Cas9 and donor plasmids, followed by infection with parental DEV. Recombinant DEV were isolated by plaque purification and confirmed by PCR.

### Virus PCR identification and stability analysis

CEF cells infected with recombinant DEV were harvested at 72 h post-infection (**hpi**). Genomic DNA was extracted using the TIANamp Genomic DNA Kit (Tiangen Biotech, Beijing, China), and PCR amplification was performed for viral identification. To evaluate genetic stability, recombinant DEV strains were serially passaged in CEF cells for 15 passages. PCR analysis was conducted every five passages using primers flanking the foreign gene insertion sites. The primers used for each locus were as follows: For the US7/US8 locus: F 5′-ATACGCTCTGGAATC ACAA-3′ and R 5′-TAGTCGCAATCCTTGTCC-3′. For the LORF11/UL55 locus: F 5′-AATCAGTCTCGGCTCTTG-3′ and R 5′-AATACGGAAACGTGGAAT-3′.

### Western blot analysis

CEF cells were infected with DEV-Vac or recombinant DEVs at an MOI of 0.01. At 48 hpi, total cellular proteins were collected and separated by 12% SDS-PAGE, followed by transfer onto polyvinylidene difluoride (**PVDF**) membranes. The membranes were incubated with monoclonal antibodies against EGFP (1:5,000), Flag (1:5,000), and β-actin (1:5,000), as well as polyclonal antibodies against DHAV VP1 (1:1,000) and DEV gC (1:1,000), followed by incubation with horseradish peroxidase (**HRP**)-conjugated secondary antibodies (1:10,000). Protein bands were visualized using an enhanced chemiluminescence (**ECL**) system and analyzed with ImageJ software.

### Growth curves of parental and recombinant DEV viruses

Replication kinetics were assessed by multi-step growth curve analysis. CEF cells were infected with DEV-Vac, rDEV-EGFP, rDEV-DHAV-VP1 or rDEV-VP1-prM/TE at an MOI of 0.01. Supernatants and cell lysates were collected at 24, 48, 72, 96, 120, and 144 hpi. Viral titers were determined using the Reed-Muench method and expressed as 50% tissue culture infectious doses (**TCID_50_**).

### Plaque size analysis

CEF cells cultured in 12-well plates were infected with DEV-Vac, rDEV-EGFP, or rDEV-VP1-prM/TE at serial dilutions. After adsorption for 2 h, the inoculum was removed and cells were overlaid with DMEM containing 1.5% methylcellulose. Following incubation at 37°C with 5% CO₂ for 48 h, plaques were visualized under an inverted fluorescence microscope. Fifty plaques from each group were randomly selected and measured using ImageJ software.

### Statistical analyses

All data were analyzed using GraphPad Prism 9.5.0 (GraphPad Software Inc., USA). Data are presented as mean ± standard deviation (**SD**). Statistical significance was defined as P < 0.05.

## Results and discussion

### Construction and characterization of recombinant rDEV-EGFP viruses

**Fig. 1:**
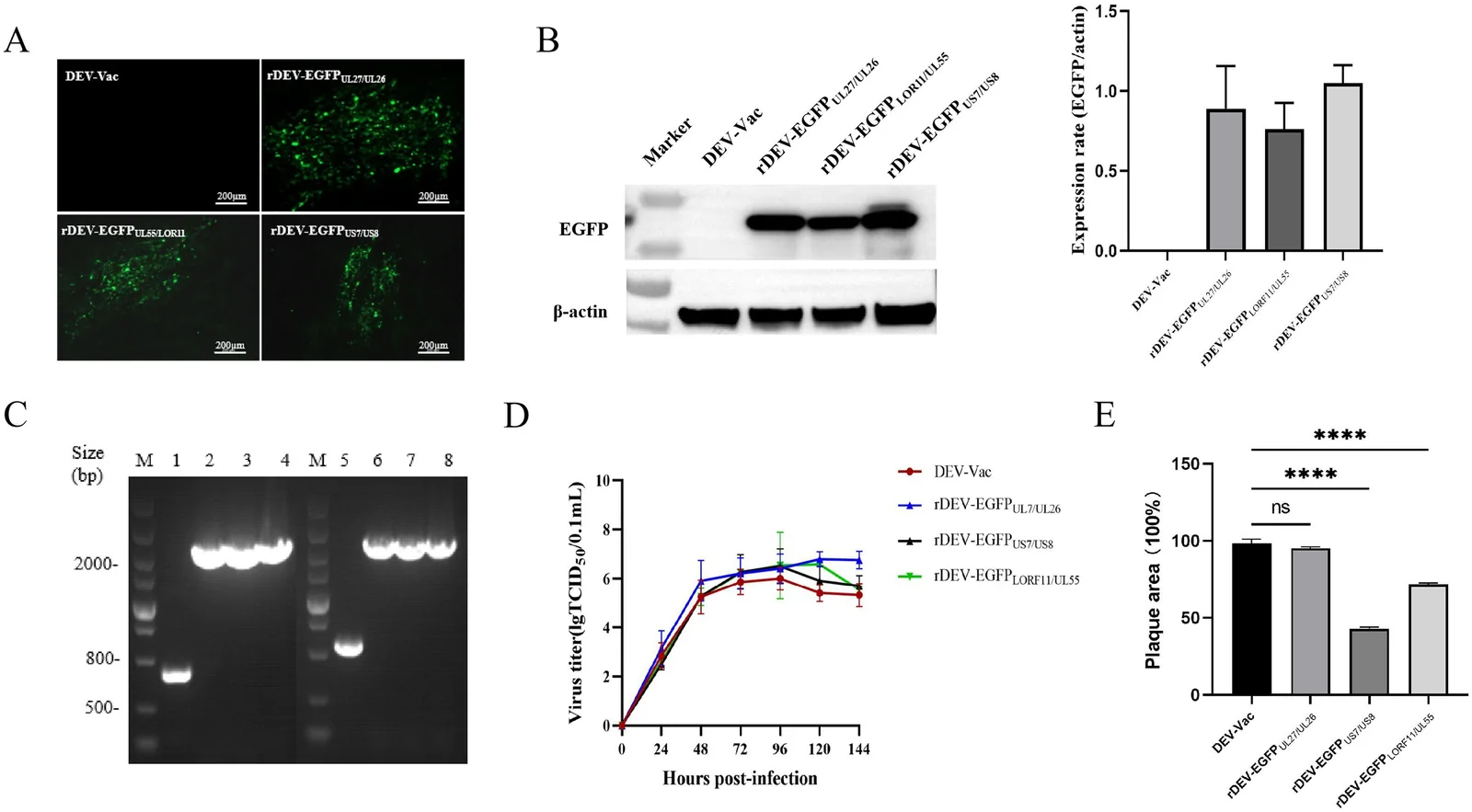
Construction and characterization of recombinant rDEV-EGFP viruses. (A) Fluorescence observation of CEF cells infected with rDEV-EGFP carrying the EGFP expression cassette at different genomic loci. (B) Western blot analysis of EGFP expression in rDEV-EGFP with different insertion loci. The left panel shows representative blots, and the right panel shows ImageJ-based densitometric analysis. (C) PCR identification of rDEV-EGFP after serial passaging. Lane M, DNA marker; lanes 1 and 5, DEV-Vac; lanes 2-4, passages 5, 10, and 15 of rDEV-EGFP_US7/US8_; lanes 6-8, passages 5, 10, and 15 of rDEV-EGFP_LORF11/UL55_. (D) Multi-step growth curves of rDEV-EGFP and parental DEV-Vac in CEF cells. (E) Plaque size analysis of rDEV-EGFP and DEV-Vac. Plaque areas were normalized to the DEV-Vac group (100%). *P < 0.05, **P < 0.01, ***P < 0.001, ****P < 0.0001.

To identify optimal insertion sites for foreign gene expression in the DEV genome, an HDR-CRISPR/Cas9-based strategy was used to insert an EGFP expression cassette into the US7/US8 and UL55/LORF11 intergenic regions of DEV-Vac. A previously constructed recombinant virus, rDEV-EGFP_UL27/UL26_, and the parental DEV-Vac were included as controls. Following plaque purification, distinct green fluorescent plaques were observed in CEF cells infected with recombinant viruses, whereas no fluorescence was detected in the DEV-Vac group (Fig. 1A), confirming successful generation of the recombinant viruses.

Western blot analysis showed that all rDEV-EGFP expressed EGFP protein. Among them, rDEV-EGFP_US7/US8_ exhibited the highest expression level, followed by rDEV-EGFP_UL27/UL26_, whereas rDEV-EGFP_LORF11/UL55_ showed relatively lower expression (Fig. 1B), indicating that insertion loci affect foreign gene expression efficiency.

To evaluate purity and genetic stability, rDEV-EGFP were serially passaged in CEF cells for 15 passages and analyzed by PCR every five passages. The expected amplicons corresponding to the inserted EGFP cassette were consistently detected at all passages, whereas DEV-Vac produced only the smaller parental band (Fig. 1C), indicating successful purification and stable maintenance of the inserted gene.

Growth curve analysis showed that all rDEV-EGFP exhibited replication kinetics comparable to those of DEV-Vac (Fig. 1D). Plaque assays revealed that rDEV-EGFP_US7/US8_ and rDEV-EGFP_LORF11/UL55_ produced significantly smaller plaques than DEV-Vac (P < 0.0001), whereas showed no significant difference was observed between rDEV-EGFP_UL27/UL26_ and DEV-Vac (Fig. 1E).

Collectively, the US7/US8, UL27/UL26, and UL55/LORF11 loci are suitable sites for foreign gene insertion in the DEV genome, as integration at these loci did not affect viral replication in vitro and remained genetically stable during serial passaging. Among them, the US7/US8 locus supported the highest level of foreign gene expression but significantly reduced plaque size.

### Construction and characterization of rDEV-VP1-prM/TE

**Fig. 2:**
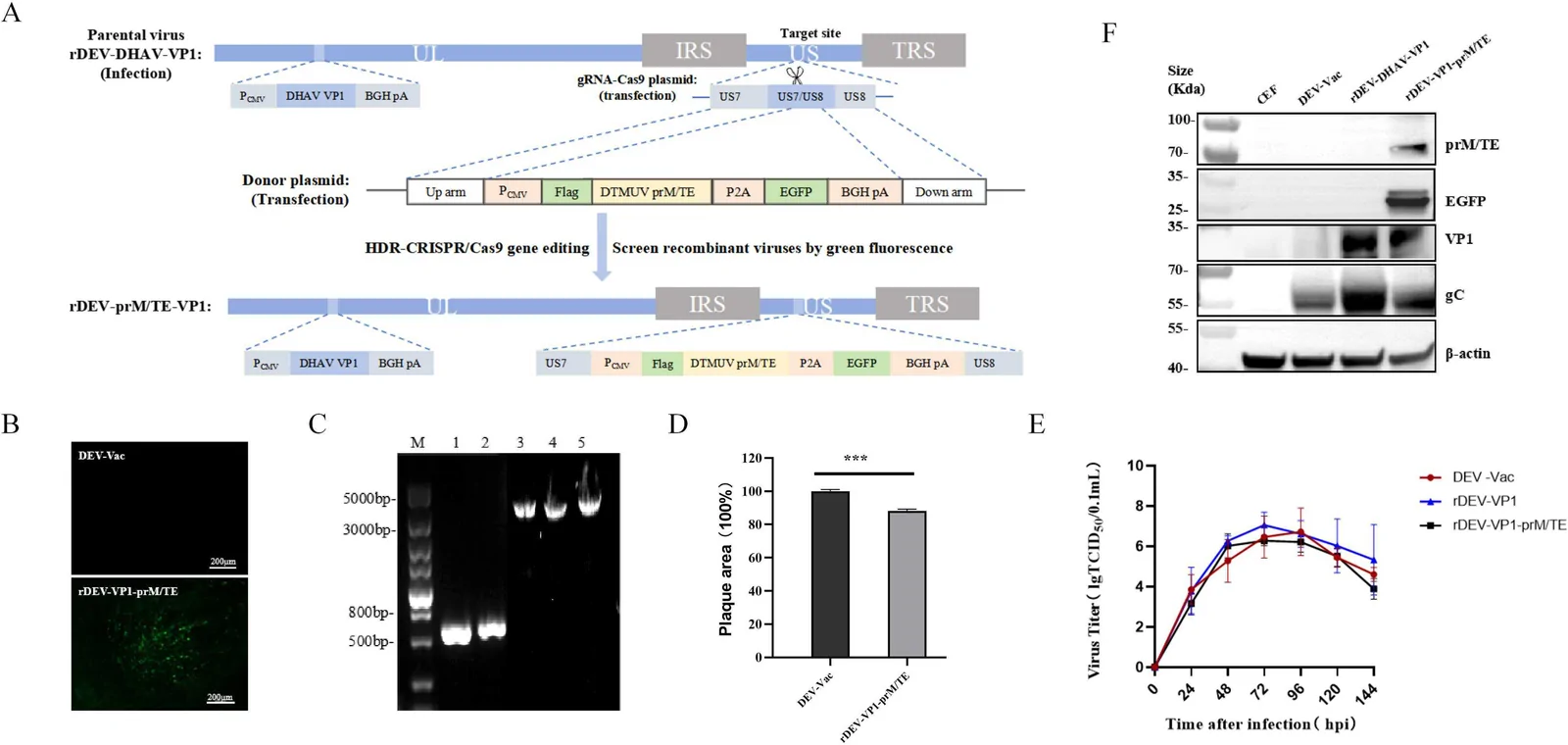
Construction and characterization of recombinant rDEV-VP1-prM/TE. (A) Schematic diagram of the construction strategy for rDEV-VP1-prM/TE. (B) Fluorescence observation of CEF cells infected with purified rDEV-VP1-prM/TE. (C) PCR identification of rDEV-VP1-prM/TE at different passages. Lane M, DNA marker; lane 1, DEV-Vac; lane 2, rDEV-DHAV-VP1; lanes 3-5, passages 5, 10, and 15 of rDEV-VP1-prM/TE. (D) Plaque size analysis of rDEV-VP1-prM/TE and parental DEV-Vac. Plaque areas were normalized to the DEV-Vac group (100%). ***P < 0.001. (E) Multi-step growth curves of rDEV-VP1-prM/TE, rDEV-DHAV-VP1, and parental DEV-Vac in CEF cells. Growth curve data were analyzed using a two-way analysis of variance (ANOVA) followed by Dunnett's multiple comparisons test. (F) Western blot analysis of foreign protein expression in rDEV-VP1-prM/TE.

Based on the previously constructed recombinant virus rDEV-DHAV-VP1, an HDR-CRISPR/Cas9-based strategy was used to insert the DTMUV prM and TE gene expression cassette into the US7/US8 intergenic region of the DEV genome, with EGFP serving as a selection marker (Fig. 2A). Following transfection-infection and plaque purification, the dual-insertion recombinant virus rDEV-VP1-prM/TE was successfully generated.

Distinct green fluorescent plaques were observed in CEF cells infected with purified rDEV-VP1-prM/TE, whereas no fluorescence signal was detected in the DEV-Vac group (Fig. 2B), confirming successful construction of the recombinant virus. PCR analysis revealed the expected amplicons in different passages of rDEV-VP1-prM/TE, while only the parental band was detected in DEV-Vac and rDEV-DHAV-VP1 (Fig. 2C), confirming successful insertion of the foreign genes and good genetic stability of the recombinant virus.

Plaque assays showed that rDEV-VP1-prM/TE produced significantly smaller plaques than DEV-Vac (P < 0.001) (Fig. 2D). However, growth curve analysis demonstrated that rDEV-VP1-prM/TE exhibited replication kinetics comparable to those of rDEV-DHAV-VP1 and DEV-Vac, with no significant differences among groups (Fig. 2E), indicating that foreign gene insertion did not markedly affect viral replication.

Western blot analysis detected specific Flag-prM/TE and EGFP protein bands in rDEV-VP1-prM/TE-infected cells but not in CEF, DEV-Vac, or rDEV-DHAV-VP1 groups (Fig. 2F), confirming expression of the Flag-prM/TE-P2A-EGFP cassette inserted at the US7/US8 locus. VP1-specific bands were detected in both rDEV-DHAV-VP1 and rDEV-VP1-prM/TE infected cells but not in CEF or DEV-Vac controls, indicating stable expression of the VP1 cassette at the UL27/UL26 locus. In addition, DEV gC protein was detected in DEV-Vac, rDEV-DHAV-VP1, and rDEV-VP1-prM/TE infected cells but not in CEF cells, confirming productive viral infection and normal expression of DEV structural proteins.

Collectively, these results demonstrate that the DTMUV prM/TE expression cassette was successfully inserted into the US7/US8 locus of rDEV-DHAV-VP1 and stably co-expressed with VP1. Although insertion of the foreign gene cassette reduced plaque size, it did not significantly affect viral replication in vitro, supporting the suitability of the US7/US8 locus for the development of multivalent DEV-vectored vaccines.

In summary, this study systematically evaluated different insertion loci in the DEV genome using an HDR-CRISPR/Cas9 strategy and demonstrated that the US7/US8 locus supported the highest foreign gene expression efficiency. Based on this locus, a recombinant DEV expressing DHAV-3 VP1 and DTMUV prM/TE genes was successfully constructed. The recombinant virus maintained stable foreign gene insertion and exhibited replication kinetics comparable to those of the parental virus in vitro. Notably, insertion of the exogenous expression cassette into the US7/US8 sites resulted in a significant reduction in viral plaque size, but did not affect the virus’s replication capacity in CEF cells, suggesting that this change may primarily affect intercellular transmission rather than the viral replication process. Whether this phenotype further affects the virus’s tissue tropism or in vivo transmissibility remains to be verified through animal studies. The results of this study provide useful information for optimization of DEV-based viral vectors and support the potential application of DEV as a multivalent vaccine platform against major duck viral diseases.

## Funding

This work was supported by the Agricultural Science and Technology Support Program of Taizhou City (No. TN202412), the Natural Science Research General Program of Jiangsu Higher Education Institutions (No. 24KJB230003), the College Students’ Innovation and Entrepreneurship Training Program (No. GX-2025-13), and Jiangsu Agri-animal Husbandry Vocational College Project (Nos. NSF2026JB01, NSFPT202516).

## Author Contribution

**Wenfeng Jia** conceived and designed the core experimental framework and the overall study, performed the major experimental work, organized and analyzed the research data, prepared the figures and data visualizations, and drafted the original manuscript. **Zhi Wu** contributed to the development of the experimental design and the interpretation of data, and played a major role in conducting the key experiments and analyzing the results. **Aiqi Tan** contributed to the acquisition, analysis, and interpretation of the experimental data, and was involved in the critical evaluation and validation of the research findings. **Yuting Cheng** contributed to the design of specific experimental procedures, performed the corresponding experimental investigations, and was involved in the interpretation of the data. **Qingkang Zhou** participated in the acquisition and interpretation of experimental data, and contributed to the verification and validation of the authenticity and reliability of the experimental results. **Shanyuan Zhu** provided overall academic guidance, contributed important intellectual insights to the study design and manuscript improvement, and supervised the progress and quality of the research. **Anping Wang** provided overarching academic guidance for the study, directed the project development, and critically revised the manuscript for crucial intellectual content.

## Disclosures

The authors declare that the research was conducted in the absence of any commercial financial relationships that could be construed as a potential conflict of interest.
